# Developmental Evaluation of an Established Advisory Board

**DOI:** 10.1093/geroni/igaa057.2860

**Published:** 2020-12-16

**Authors:** Rachel Lessem

**Affiliations:** CJE SeniorLife, Chicago, Illinois, United States

## Abstract

CJE SeniorLife developed the PCORI-funded Bureau of Sages, a research advisory board of nursing home community members and older adults, who live at home and receive long term services and supports (LTSS). Bureau members share experiences, build knowledge, and develop skills for working together to provide voice to the direction, design, and implementation of aging research. The PCORI-funded Sages in Every Setting project developed four additional bureaus, expanding and diversifying a pool of researchers, providers, and older adults living with LTSS who have the knowledge, skills, and opportunity to collaborate in research. Evaluation results reveal that the Sage Model is translatable into new settings; partners were able to adapt the provided resources; researchers receive useful input from bureaus to improve their work; and, participation in a research advisory board is a benefit to its members. In addition, we will share challenges to establishing and expanding the Sage Model. Part of a symposium sponsored by the Patient/Person Engagement in Research Interest Group.

